# Cerebrospinal fluid microRNAs are potential biomarkers of temporal lobe epilepsy and status epilepticus

**DOI:** 10.1038/s41598-017-02969-6

**Published:** 2017-06-12

**Authors:** Rana Raoof, Eva M. Jimenez-Mateos, Sebastian Bauer, Björn Tackenberg, Felix Rosenow, Johannes Lang, Müjgan Dogan Onugoren, Hajo Hamer, Tessa Huchtemann, Peter Körtvélyessy, Niamh M. C. Connolly, Shona Pfeiffer, Jochen H. M. Prehn, Michael A. Farrell, Donncha F. O’Brien, David C. Henshall, Catherine Mooney

**Affiliations:** 1 Department of Physiology & Medical Physics, Royal College of Surgeons in Ireland, 123 St Stephen’s Green, Dublin 2, Ireland; 2 Department of Anatomy, Mosul Medical College, University of Mosul, Mosul, Iraq; 3 Epilepsy Center Hessen, Department of Neurology, Baldingerstr, 35043 Marburg, Germany; 4 Epilepsy Center Frankfurt Rhine-Main, Neurocenter, Goethe-University, Schleusenweg 2-16, Haus 95, 60528 Frankfurt a.M., Germany; 5 University Hospital Erlangen, Schwabachanlage 6, 91054 Erlangen, Germany; 6 Department of Neurology, University Hospital Magdeburg, Leipziger Strasse 44, 39120 Magdeburg, Germany; 7 Beaumont Hospital, Beaumont Road, Dublin 9, Ireland

## Abstract

There is a need for diagnostic biomarkers of epilepsy and status epilepticus to support clinical examination, electroencephalography and neuroimaging. Extracellular microRNAs may be potentially ideal biomarkers since some are expressed uniquely within specific brain regions and cell types. Cerebrospinal fluid offers a source of microRNA biomarkers with the advantage of being in close contact with the target tissue and sites of pathology. Here we profiled microRNA levels in cerebrospinal fluid from patients with temporal lobe epilepsy or status epilepticus, and compared findings to matched controls. Differential expression of 20 microRNAs was detected between patient groups and controls. A validation phase included an expanded cohort and samples from patients with other neurological diseases. This identified lower levels of miR-19b in temporal lobe epilepsy compared to controls, status epilepticus and other neurological diseases. Levels of miR-451a were higher in status epilepticus compared to other groups whereas miR-21-5p differed in status epilepticus compared to temporal lobe epilepsy but not to other neurological diseases. Targets of these microRNAs include proteins regulating neuronal death, tissue remodelling, gliosis and inflammation. The present study indicates cerebrospinal fluid contains microRNAs that can support differential diagnosis of temporal lobe epilepsy and status epilepticus from other neurological and non-neurological diseases.

## Introduction

Temporal lobe epilepsy (TLE) is the most common intractable form of epilepsy in adults, with seizures originating from or involving mesial temporal structures such as the hippocampus. Seizures are the result of abnormal excessive neuronal discharges in the brain and can produce symptoms ranging in severity from a brief sensory experience to a major convulsive episode^1^. When seizures cannot be terminated by inhibitory mechanisms, status epilepticus (SE) results, which is one of the most frequent neurological emergencies with an incidence of about 20/100,000, potential to cause brain injury after 30–60 min, and a case fatality rate of around 15%^2, 3^. The diagnosis of both conditions remains principally based on clinical assessment, including a detailed patient history. This history may be incomplete however, and seizures are often not witnessed by the treating physician. EEG and neuroimaging are frequently required to make a definite diagnosis but require specialist clinical staff and equipment which are not available to many patients. It is estimated that up to 30% of patients are mis-diagnosed with epilepsy^4, 5^. Frequent causes of misdiagnosis are psychogenic non-epileptic seizures and syncope^4, 6^. Furthermore, patients with confusion or coma of unknown aetiology frequently have non-convulsive SE, which can only be detected by EEG and is therefore frequently overlooked^7–9^.

A molecular biomarker that could distinguish epilepsy and SE from other neurological conditions would allow for an earlier and more accurate diagnosis and appropriate treatment^10, 11^. While there is not a clinical diagnostic demand for a biomarker to discriminate patients with SE from patients with TLE there would be pathophysiologic and mechanistic value to knowing how prolonged individual seizures and repeated brief seizures affect biomarker profiles. Such a molecule would need to combine sensitivity and specificity with suitable physico-chemical properties such as stability in body fluids, be amenable to rapid, reliable measurement and demonstrate mechanistic links to causal patho-mechanisms of epilepsy and prolonged seizures^10, 11^. MicroRNAs are small endogenous, non-coding RNA molecules which regulate gene expression in a post-transcriptional manner by binding to complementary sequences in mRNAs^12^. To function, microRNAs are first uploaded to the RNA-induced silencing complex which contains the protein Argonaute2. The microRNA is then guided to the target mRNA resulting in either degradation of the mRNA or a reduction in translation to protein^13^. Extracellular microRNAs are a promising class of biomarker and altered levels of circulating microRNAs have been reported in various human diseases^14, 15^. Their presence is presumed to be a consequence of passive release from damaged cells as well as active (paracrine signalling) mechanisms^16^. Once released, microRNAs circulate in different forms including bound to Argonaute2 and enclosed within microvessicles such as exosomes^17–19^. This provides stability and microRNAs can be measured using straightforward microarray, polymerase chain reaction or sequencing-based techniques^20^.

The brain expresses the largest variety of microRNAs of any organ and unique microRNAs are found in different brain regions and in neurons, astrocytes and other cell types^21, 22^. Altered expression of more than 1,000 different microRNAs has been reported within the hippocampus and other involved brain structures in experimental and human epilepsy and SE^23^. Functional studies have demonstrated that targeting microRNAs can alter brain excitability and produce or inhibit evoked and spontaneous seizures and SE^24^. Recently, blood levels of a number of microRNAs have been reported to be altered during specific phases of epilepsy development in rodent models and in patients with epilepsy and SE^25–29^. This includes elevated levels of miR-21-5p and miR-146a-5p after experimental status epilepticus and elevated levels of miR-146a-5p and miR-194-5p in serum from patients with epilepsy.

Cerebrospinal fluid (CSF) is a potential source of microRNA biomarkers of epilepsy and SE, with the advantage over plasma or serum of being in more direct contact with the extracellular space of the target tissue or region of pathology^20^. MicroRNAs in CSF demonstrate suitable stability when exposed to repeated freeze-thaw cycles and long-term storage^30^ and altered microRNA expression has been reported in CSF from patients with Alzheimer’s disease^31^, multiple sclerosis^32^ and malignancies^30^. Repetitive or prolonged seizures can provoke neuronal injury, activation of glia and interruption of the integrity of the blood- or CSF-brain barrier that would be expected to alter the microRNA content of CSF. The aim of this study was to investigate microRNA expression profiles in the CSF of patients with TLE and SE and compare these to other neurological diseases to explore their role as potential biomarkers which would assist clinicians when making the diagnosis.

## Materials and Methods

### Patients

CSF samples were collected from three different centres during clinical workup: the University of Magdeburg, The Philipps University of Marburg and The Friedrich-Alexander-University Erlangen-Nurnberg. Consent was obtained according to the Declaration of Helsinki and ethical approval was obtained from the local medical ethics committees at each centre. This included informed written consent given by patients or their legal representatives if the patients were obtunded. The study was conducted in two phases: a discovery/profiling phase and a validation phase.

#### Discovery/profiling

Forty-five adults were recruited, divided into three groups. The control group samples comprised CSF collected from patients who presented with headache to exclude infectious causes which was confirmed negative (*n* = 15; CH). The other samples were from TLE patients (*n* = 15) and patients who experienced SE (*n* = 15). Clinical details are summarised in Table 1 (and see Supplementary Table S1 for full details).

**Table 1:** Summary of patient group demographics.

	CH	TLE	
**M**	10 (29.8 ± 2.2 yrs)	9 (35.8 ± 5 yrs)	
**F**	18 (39.9 ± 2 yrs)	6 (46.3 ± 3 yrs)	
	**SE**		
	**FSE**	**GTC**	**NCSE**
**M**	4 (72.2 ± 7.3 yrs)	3 (76 ± 4.7 yrs)	3 (68 ± 10.4 yrs)
**F**	6 (72.8 ± 4.7 yrs)	n/a	2 (63.5 ± 10.5 yrs)
	**CND**		
	**AD**	**MS**	**others**
**M**	6 (73.3 ± 2.7 yrs)	2 (62 ± 19 yrs)	3 (74.6 ± 2.1 yrs)
**F**	3 (72 ± 3.6 yrs)	8 (32.3 ± 2 yrs)	3 (60.6 ± 3.8 yrs)

*Key;* AD, Alzheimer’s disease; CH, control (headache); F, female; FSE, focal status epilepticus; GTC, generalized tonic clonic seizures; M, male; MS, multiple sclerosis; n/a; not applicable; NCSE, nonconvulsive status epilepticus; SE, status epilepticus; TLE, temporal lobe epilepsy.

#### Validation

MicroRNA expression was validated in CSF samples from 25 controls, 14 TLE and 17 SE patients. A fourth group of 25 patients diagnosed with other neurological diseases (CND) was included in the validation phase as a second control group (see Table 1 and Supplementary Table S1). This allowed us to check if microRNA dysregulation is specific for TLE or SE, or a more unspecific marker of neurological diseases in general. The majority of these samples were from patients with Alzheimer’s disease and multiple sclerosis due to the more common sampling of this biofluid from these patients. We were unable to obtain sufficient samples from patients with psychogenic non-epileptic seizures or syncope for the present study.

### Biofluid collection

CSF was centrifuged within one hour of collection at 300 × g for 10 min at 4 °C to remove contamination or cellular debris, the supernatant was collected and stored at −80 °C until further use. Plasma samples were collected from a subgroup of TLE patients on the same day as CSF collection. 5 ml of peripheral blood was collected and plasma was prepared by centrifuging the tubes at 1300 × g, for 10 min, at 4 °C and stored at −80 °C. A second centrifugation step was performed at 1940 × g for 10 min at 4 °C to further reduce cellular contamination^33^. Absorbance at 414 nm was checked using Nanodrop 2000 spectrophotometer, and any samples with possible haemolysis (414 absorbance >0.2) were excluded^34^.

### RNA extraction and microRNA expression profiling

Total RNA was extracted from 200 μl of CSF or plasma using the miRCURY RNA isolation kit (Exiqon) according to the manufacturer’s protocol. MicroRNA profiling was performed using the QuantStudio 12 K Flex TaqMan OpenArray System (ThermoFisher Scientific). This detects over 750 mature human microRNAs. RNA extraction and microRNA profiling protocols were as described^35^ (and see Supplementary Methods).

### Reverse transcription and real-time PCR validation

Validation of OpenArray findings was performed using Taqman small-scale microRNA assays (Applied Biosystems) as described^36, 37^. The amplification was performed in triplicate and a negative control was included for each primer. Real time qPCR was carried out on a 12 K Flex Quant-Studio PCR system. The same protocol was followed to quantify the microRNA levels of miR-19b-3p, miR-21-5p and miR-451a in plasma samples from TLE patients.

### Digital PCR

Digital PCR (dPCR) was performed to obtain estimates of microRNA copy number in CSF. A set of three representative CSF samples from each group were selected. Briefly, total RNA was reverse transcribed using microRNA-specific RT primers following the same protocol used for individual RT-qPCR. The generated cDNA was used as a template for dPCR using the QuantStudio 3D Digital PCR System (ThermoFisher). First, to determine the optimal digital range of dPCR reaction, cDNA was serially diluted over the range 1:2, 1:4, 1:8, 1:16 and 1:32. A reaction mix was prepared containing dPCR master mix, microRNA-specific TaqMan assays and diluted cDNA and loaded onto individual QuantStudio v2 dPCR chips. Each chip contains 20,000 individual wells, within which cDNA is randomly and uniformly distributed. Diluting cDNA by 1:2 allowed each well to contain either zero or one target molecule only. The PCR was performed in a Proflex 2× flat block thermal cycler (ThermoFisher) using standard conditions: 96 °C for 10 min; 39 cycles of 60 °C for 2 min and 98 °C for 30 sec and 60 °C for 2 min. Chips were then primarily processed using the QuantStudio 3D instrument to obtain the number of wells with an amplified target (FAM positive) and number of empty wells (FAM negative). Copy numbers were determined using the QuantStudio 3D AnalysisSuite Cloud software.

### Analysis of Argonuate2 and exosome microRNAs in CSF

To analyse microRNA levels complexed to Argonaute2 or bound in exosomes, samples from each group were pooled into five pools each. For other neurological diseases we generated three pools of multiple sclerosis samples and three for the Alzheimer’s disease samples. Total RNA, Argonaute2 and exosomes were extracted and precipitated from each pool. Exosome precipitation was performed using the ExoQuick Exosome Precipitation Solution (System Biosciences-SBI)^38^. Exosomal RNA was then extracted as above. Argonaute2 pull-down from samples was adapted as described^18, 36^. RNA was extracted from Argonaute2 immunoprecipitates using Trizol reagent (Invitrogen). Levels of miR-19b-3p, miR-21-5p and miR-451a were measured using RT-qPCR and a preamplification step included to increase level of detection.

### *In situ* hybridization detection of microRNA

Formalin-fixed, paraffin-embedded hippocampal tissue sections were obtained after surgical resection from TLE patients or as postmortem tissue sections from patients who died from severe intractable SE. *In situ* hybridization was performed as previously described^36^ with adaptations. Briefly, miR-19b-3p, miR-21-5p and miR-451a signals were localized after an overnight incubation with digoxigenin (DIG) labelled, LNA detection probes (Exiqon). Secondary detection of the probe-miR interaction was performed using anti-Dig antibody (Anti-DIG-POD Fab fragment) (Roche). Tyramide signal amplification plus fluorescence (Cy3) kit (Perkin Elmer) was used to amplify the *in situ* labelling of the target microRNA. Fluorescence labelling of astrocytes was performed using antibodies against the astrocyte marker, glial fibrillary acidic protein (GFAP, Sigma-Aldrich Ireland) and AlexaFluor488 secondary antibodies (ThermoFisher Scientific). Fluorescent signals were imaged using LSM 710 confocal laser scanning microscope (Zeiss).

### Pathway analyses and bioinformatics

Experimentally validated microRNA targets for miR-19b-3p, miR-21-5p and miR-451a were retrieved from miRTarBase^39^. Only human targets with strong evidence were retained (reporter assay, western blot, qRT-PCR or qPCR; Table S2), and a microRNA-target interaction network was generated using Cytoscape^40^. We uploaded the genes identified as validated targets to Enrichr (gene set enrichment analysis web server)^41^ and explored the gene ontology biological processes, molecular function and pathway involvement of the targets. The significantly associated gene ontology terms (adjusted p-value < 0.05) were imported to REVIGO where they were clustered based on their relatedness and any redundancy was removed^42^. Significantly enriched PANTHER pathways^43^, with adjusted *p*-value < 0.001 were exported from Enrichr and plotted in Microsoft Excel.

Validated targets of miR-19b-3p, miR-21-5p and miR-451a were also compared with validated human targets of 15 additional microRNAs previously implicated in epilepsy (miR-22-3p, miR-23b-5p, miR-34a-5p, miR-124-3p, miR-128-3p, miR-132-3p, miR-134-5p, miR-146a-5p, miR-155-5p, miR-184, miR-199a-5p, miR-203a-3p, miR-210-3p, miR-219a-5p, miR-324-5p), which each have *in vivo* functional data showing effects on seizures and/or pathology in rat or mouse epilepsy models^23, 24, 44^. Finally, we cross-checked the validated targets against two databases of epilepsy gene mutations: CARPEDB (http://carpedb.ua.edu/) and epiGAD(http://www.epigad.org)^45^.

### Data and statistical analysis

OpenArray profiling data were analysed in two steps. First, to ensure good quality detection and to avoid false-positives the data were filtered according to “AmpScore” or “CqConf” values provided by the ExpressionSuite software (ThermoFisher Scientific). Cutoff thresholds were set to 35. This gave us a general idea about microRNA expression in CSF. Second, more in depth data analysis was performed in R/Bioconductor^46, 47^ and a more stringent “present”/“absent” filtering step was included whereby a microRNA was considered “present” in a set of samples if the Ct was <28 in 60% of the samples. A microRNA was considered “absent” if the Ct was >28 in 80% of the same samples. MicroRNAs were removed unless they were either present in both control/TLE or control/SE samples, or if they were absent from one set of samples and present in the other. Missing data points were imputed (Bioconductor package “non-detects”^48^), the data was normalised to the geometric mean (GM) as implemented in Bioconductor package “HTqPCR”^49^ and corrected for batch effects due to sample origin (Bioconductor package “ComBat”^50^). Differential expression analysis was performed using the *limma* package^51^. *P*-values were adjusted for multiple testing by controlling the false discovery rate (FDR) according to the method of Benjamini and Hochberg^52^. A microRNA was considered to be differentially expressed if the adjusted *p*-value was <0.05. Fold changes (FC) were calculated as FC = 2^−∆Ct^, where ∆Ct = Ct_microRNA_ − GM.

A number of differentially expressed microRNAs were selected for validation using individual RT-qPCR assays. MicroRNA levels were normalised to miR-24 (as reported^30^), using the ∆Ct method. The mean of the Ct for a particular microRNA were calculated after removing outliers identified with Grubbs’s test (*p*-value ≤ 0.05). ANOVA followed by Tukey’s post-hoc test was carried out to test for any significant differences between the means. A microRNA was considered to be differentially expressed if the adjusted *p*-value was ≤0.05. Fold changes were calculated as FC = 2^−∆∆Ct^ where ∆∆Ct = ∆Ct_target_ − ∆Ct_ref_. Relative expression of Argonaute2 bound and exosomal microRNA relative to total microRNA expression in samples was calculated as 2^(miR-Total − miR-AGO)^ and 2^(miR-Total − miR-EXO)^ respectively. Correlations were determined by computing Spearman’s rank correlation coefficient (*ρ*). Receiver operating characteristic (ROC) curve analyses were performed using the R pROC package^53^. Logistic regression analysis of the combined microRNA was carried out with the R glm package using the normalised expression of microRNAs from qRT-PCR experiments as independent variable, and TLE or SE status as the dependent variable. We used the miRNAmeConverter Bioconductor package^54^, to convert microRNA names from the miRBase V14 (used on the OpenArray platform) to miRBase V21.

## Results

### Profiling microRNAs in CSF from TLE and SE patients

**Figure 1:**
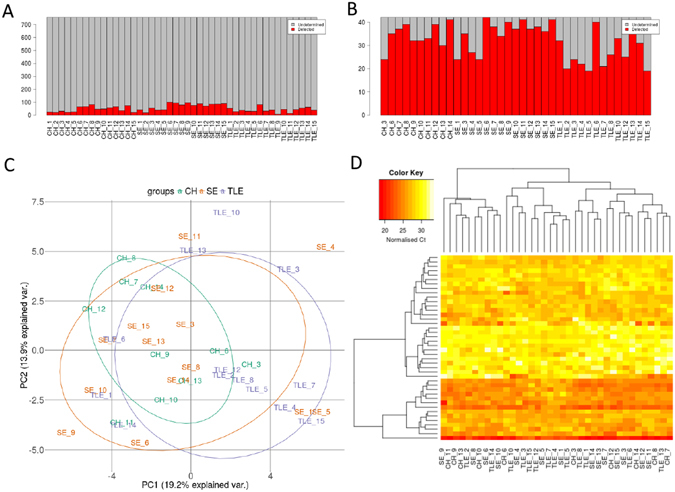
Bar plot showing the number of microRNA detected in each sample (**A**) before and (**B**) after filtering to remove any microRNA not present in at least 50% of samples. (**C**) Principal component analysis (PCA) plot. The PCA was performed on samples after imputing missing values, normalisation of Ct values and batch correction for sample origin. (**D**) Heat map and unsupervised two-way hierarchical clustering by sample and microRNA. Dendrograms were constructed using complete linkage method with Euclidean distance measure.

An average of 51 microRNA were detected per CSF sample from microRNA profiling using the OpenArray platform (range: 6–100) (Fig. 1A). Eight samples had fewer than 25 microRNAs identified (five controls, two TLE and one SE sample) and these were removed, leaving 37 samples (10 controls; 13 TLE; 14 SE). In CSF from TLE patients we detected similar numbers of microRNA to the control group (43 and 56 on average per samples respectively). A higher number of microRNAs were present in the SE samples (average 74 per sample, p < 0.001). Forty-two microRNAs were identified in at least 50% of all samples (Ct < 35) (Fig. 1B). The most abundant were: miR-19b-3p, miR-24-3p, miR-30b-5p, miR-30c-5p, miR-150-5p, miR-204-5p, miR-223-3p, miR-320a and miR-483-5p. Overall, the most abundant and most consistently detected microRNA in each of the three sets of samples were generally very similar. A principal component analysis did not show any clear separation of the three groups (Fig. 1C). A heatmap showing hierarchical clustering of the normalised Ct values in the samples in two dimensions again confirmed this lack of clustering of the samples (Fig. 1D). This would suggest that overall the CSF microRNA profiles of TLE and SE patients are broadly similar to controls. Although principal component analysis and clustering did not reveal large differences in microRNA profiles between the groups we nevertheless detected a number of significantly regulated microRNAs. This included five differentially expressed in TLE compared to controls (one down and four up regulated) and 15 microRNAs in SE samples compared to controls (seven down and eight up regulated) (Table 2).

**Table 2:** microRNA differentially expressed in profiling between patient groups and controls.

CH and SE samples		
microRNA	log_2_(FC)	adj *p*-value
miR-21-5p*	18.63	<0.001
miR-451a*	18.29	<0.001
miR-886-3p*	−5.97	<0.001
miR-9-3p	4.63	<0.001
miR-17-5p	9.9	<0.001
miR-206	5.69	<0.001
miR-92a-3p	2.08	<0.001
miR-20a-5p	6.92	<0.001
miR-204-5p*	−2.12	0.001
miR-30c-5p	−1.82	0.001
miR-331-3p	−1.61	0.006
miR-376a-3p	−2.04	0.01
miR-19b-3p*	1.52	0.017
miR-30b-5p	−1.02	0.021
miR-99b-5p	−1.19	0.034
**CH and TLE samples**		
miR-19b-3p*	0.94	0.03
miR-204-5p*	−1.71	0.03
miR-223-3p*	1.2	0.03
miR-328-3p	0.97	0.03
miR-548a-3p	1.6	0.03

Key: CH, control headache; SE, status epilepticus; TLE, temporal lobe epilepsy. log_2_(FC) and adjusted *p*-value for significantly differential expressed microRNA (OpenArray). *MicroRNA selected for validation.

### Validation of differentially expressed microRNAs in CSF from TLE and SE patients

**Figure 2:**
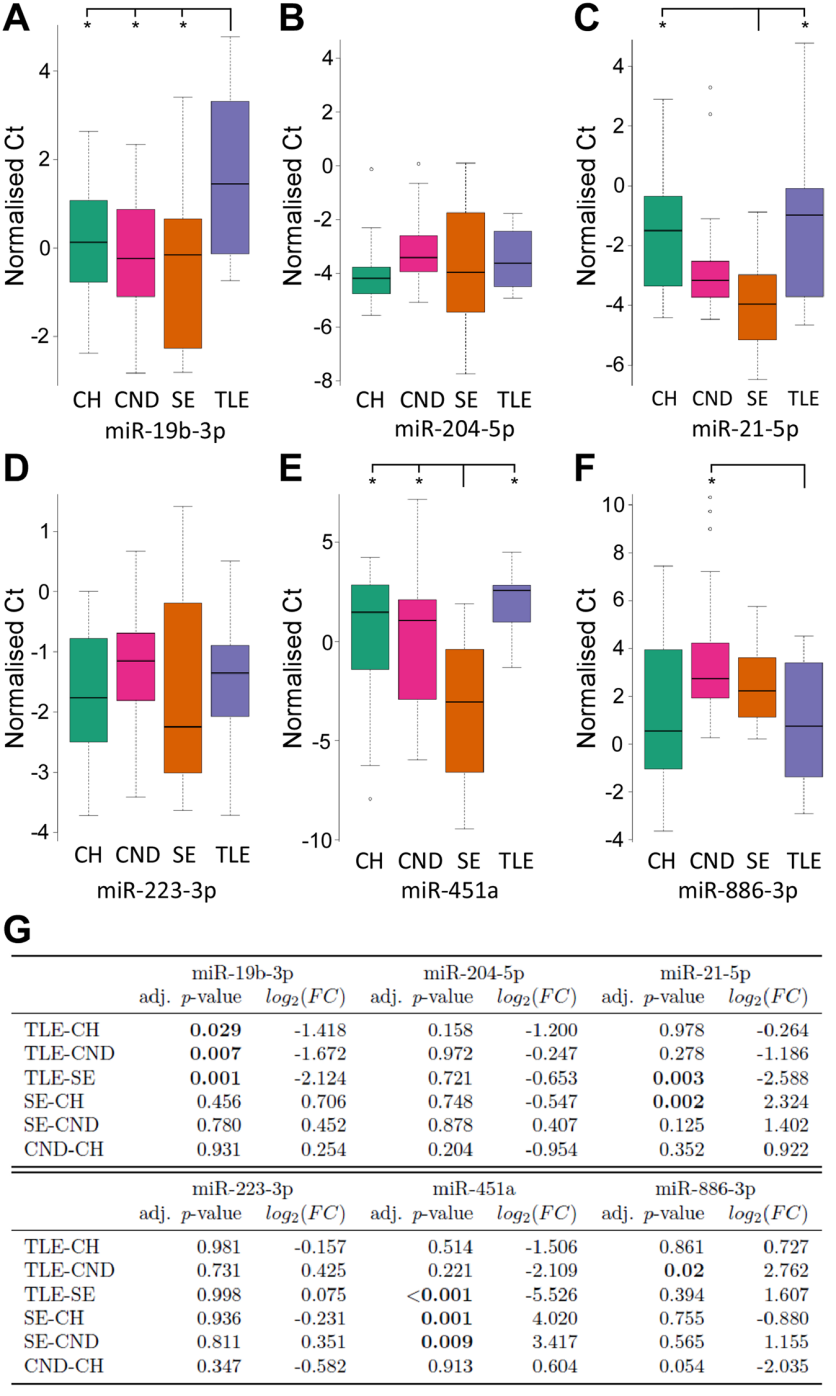
Validation of microRNA. (**A**–**F**) Boxplots showing the distribution of normalised Ct following Taqman individual microRNA assays of miR-19b-3p, miR-21-5p, miR-204-5p, miR-223-3p, miR-451a and miR-886-3p in each of the four sets of samples: CH (N = 25), CND (N = 25), SE (N = 16) and TLE (N = 14). Note, the data shown are normalized and the lower the value the greater the relative expression of the microRNA. Outliers not graphed for purposes of data presentation are shown as circles. (**G**) log_2_(FC) and adjusted *p*-value for validated microRNA. CH: controls with headache, TLE: temporal lobe epilepsy, SE: status epilepticus, CND: controls with other neurological diseases.

For the validation of the microRNA profiling we selected three microRNAs with the highest fold change and lowest *p*-values: miR-21-5p, miR-451a and miR-886-3p to validate with individual RT-qPCR. We also selected miR-204-5p and miR-19b-3p as they were significantly differentially expressed in both control/TLE and control/SE. Finally, as the significantly differentially expressed microRNAs between the control and TLE samples all had borderline significant *p*-value, and none of them had a log_2_-fold change above 2, we chose miR-223-3p as it has been shown to be expressed in astrocytes and microglia^22^. During the validation step we included a fourth group of samples from patients with other neurological diseases. With the exception of miR-19b-3p, the validation experiments confirmed the OpenArray profiling data and statistical analysis showed a number of the microRNA to be significantly differentially expressed between these four groups (Fig. 2A–G and Supplementary Fig. S1).

Validation determined that among the three microRNAs found to be differentially expressed between control and TLE during profiling, levels of miR-19b-3p were confirmed as significantly differentially expressed (Fig. 2A). Levels of miR-19b-3p were significantly lower in TLE samples compared to controls, SE and samples from patients with other neurological diseases, indicating reduced CSF levels of miR-19b-3p may be a biomarker of TLE.

Validation also identified a CSF microRNA that was specific for SE. Levels of miR-451a were higher in SE compared to controls, TLE and other neurological disease samples (Fig. 2E,G). Expression of miR-21-5p was also significantly higher in SE samples compared to TLE samples and the control group, suggesting this may be a non-specific biomarker of brain metabolism due to seizure activity (Fig. 2C,G). Although miR-21-5p was at higher levels in SE compared to other neurological diseases, this was not significant. Levels of miR-886-3p, which had been found down-regulated in SE compared to controls during profiling was significantly lower during validation in TLE samples compared to samples from patients with other neurological diseases (Fig. 2F,G). The level of miR-886-3p was also borderline significant between the two control groups (controls and other neurological diseases). The levels of miR-204-5p and miR-223-3p were not significantly differentially expressed between groups.

### Assessment of microRNA copy number in CSF

**Figure 3:**
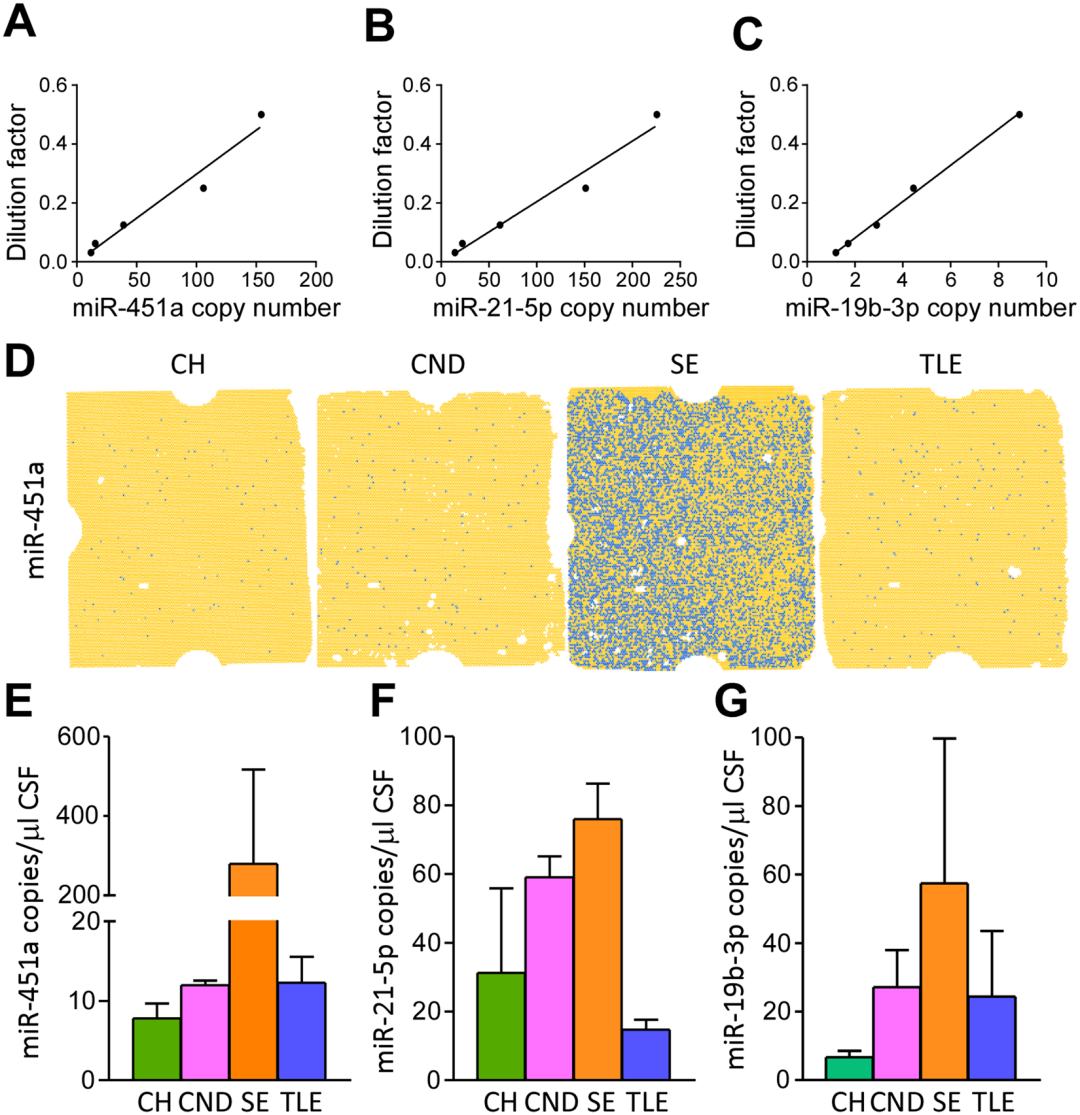
Determination of microRNA copy number in CSF. (**A**–**C**) Graphs depicting detection of miR-451a, miR-21-5p and miR-19b-3p per µl of dPCR reaction assay in serially diluted cDNA samples confirming suitability for measuring microRNA copy numbers in CSF. (**D**) Representative chips show the number of miR-451a positive wells in cDNA isolated from CSF samples for each group. Blue dots represent positive signal from amplified miR-451a. (**E**,**F**) Copy numbers for miR-451a, miR-21-5p and miR-19b-3p in representative CSF samples from the four groups (*n* = 3/group).

To extend the validation of the three microRNAs we used digital PCR (dPCR) to obtain an estimation of actual copy number within the CSF (Fig. 3). The dynamic range of the dPCR was first determined using serial dilutions of cDNA. This showed excellent linearity between the cDNA input and copy number values measured for miR-451a, miR-21-5p and miR-19b-3p (Fig. 3A–C) confirming the suitability of dPCR for detecting microRNA copy numbers in CSF samples. The absolute microRNA copy number was then calculated in a representative set of three CSF samples from each group. Figure 3D shows a representative dPCR chip result for miR-451a in a sample from each group. MicroRNA copy number in CSF was highest for miR-451a in SE samples, with an average of ~280 copies per µl CSF (range 26–745). Copy number of miR-451a was low in samples from the other three groups (Fig. 3E). Copy numbers for miR-21-5p also followed the trend expected from the profiling and validation studies with highest numbers in SE samples followed by CND samples (Fig. 3F). The average copy number of miR-19b-3p determined by dPCR did not follow as closely with the profiling and validation, although levels in TLE samples were lower than in the SE samples as expected (Fig. 3G).

### Influence of age on CSF levels of microRNAs

Since our SE group included, on average, older patients than our TLE and control groups we checked whether there were age-related influences on the expression of the validated microRNAs in CSF. We thus plotted relative expression of the four microRNAs against patient age (Supplementary Fig. S2). While there was no influence of age on CSF levels of miR-19b-3p, miR-21-5p or miR-451a, there was a strong correlation for miR-886-3p indicting the dysregulation of miR-886-3p could be biased by an effect of age. We therefore dropped miR-886-3p from the remaining analyses.

### microRNA as predictive biomarkers

Receiver operating characteristic (ROC) analysis was performed to evaluate the performance of miR-19b-3p, miR-21-5p and miR-451a to discriminate TLE and SE samples from each other and the two control groups. The closer the value of the area under the curve (AUC) to 1 the better the sensitivity and specificity of the microRNA to discriminate between groups. Supplementary Figures S3, S4 and S5 show sensitivity plotted against specificity for the significantly differentially expressed microRNA in each of the contrasts. The best performing single microRNA was miR-451a with an area AUC of 0.91 for separation between TLE and SE samples. miR-21-5p also showed good separation between SE and control samples with an AUC of 0.83. Levels of miR-19b-3p showed good separation between TLE and each other sample type of between 0.73 and 0.78.

**Figure 4:**
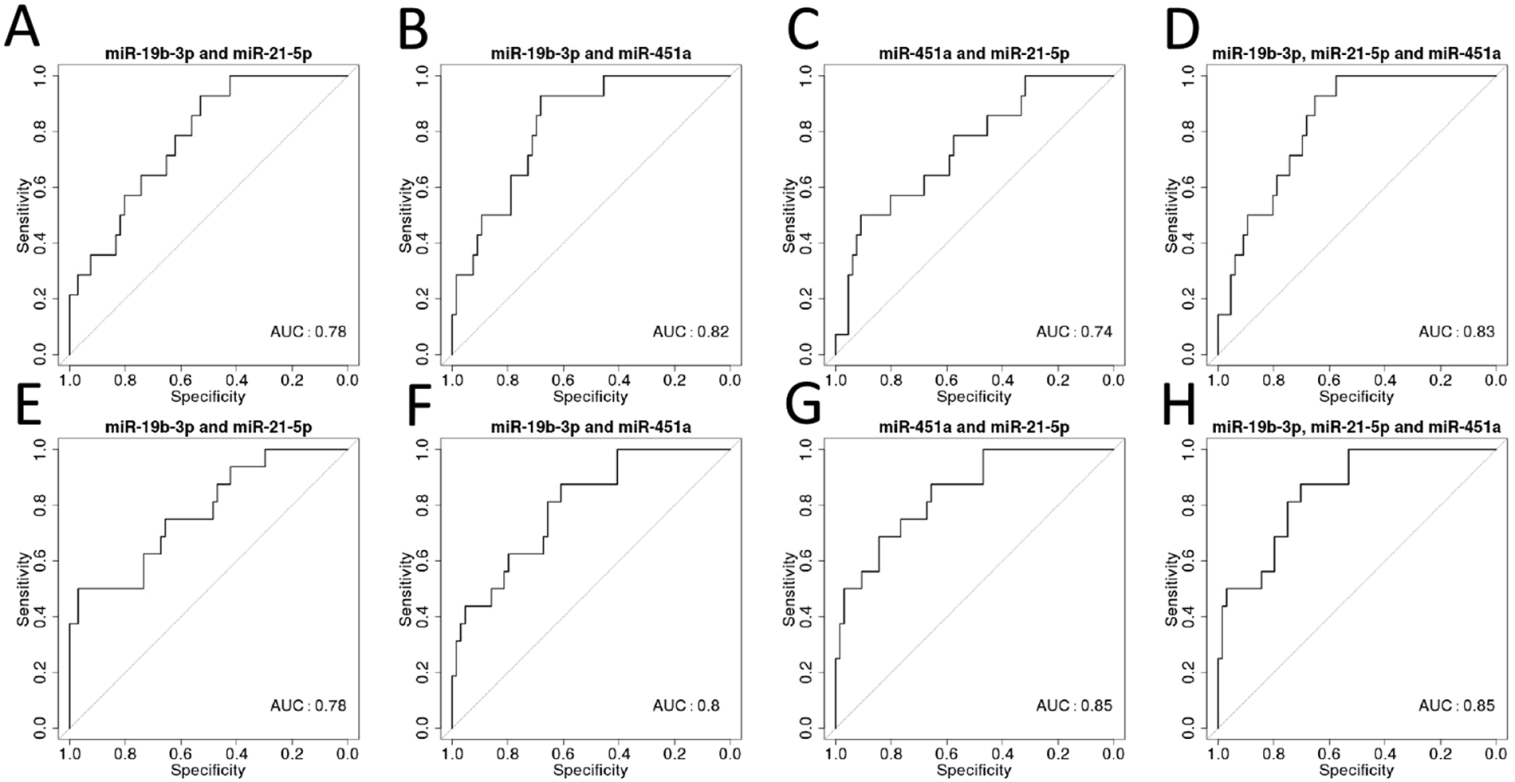
ROC analysis using binomial logistic regression to combine microRNAs for (**A**–**D**) TLE samples versus CH, CND and (**E**–**H**) SE samples versus CH, CND and TLE samples.

Binomial logistic regression was then performed to identify the best discriminating combinations of microRNAs. By combining all three microRNAs we were able to achieve an AUC of 0.83 for TLE samples compared to all other groups, compared to the best single microRNA (miR-19b-3p, AUC = 0.75) or pair of microRNAs (miR-19b-3p and miR-451a, AUC = 0.82) (Fig. 4A–D). However, when the comparison was made between SE and other groups, the combination of miR-21–5p and miR-451a (AUC = 0.85) was as accurate as all three microRNA combined (Fig. 4E–H).

Collectively, our results suggest that CSF miR-19b-3p, miR-21-5p and miR-451a may be a novel combination of microRNAs which can discriminate TLE samples from controls, patients with other neurological diseases and SE. The results also show that the combination of two microRNAs (miR-21-5p and miR-451a) can distinguish SE samples from TLE, controls and other neurological diseases.

### Extracellular transport mechanisms of microRNAs in CSF

**Figure 5:**
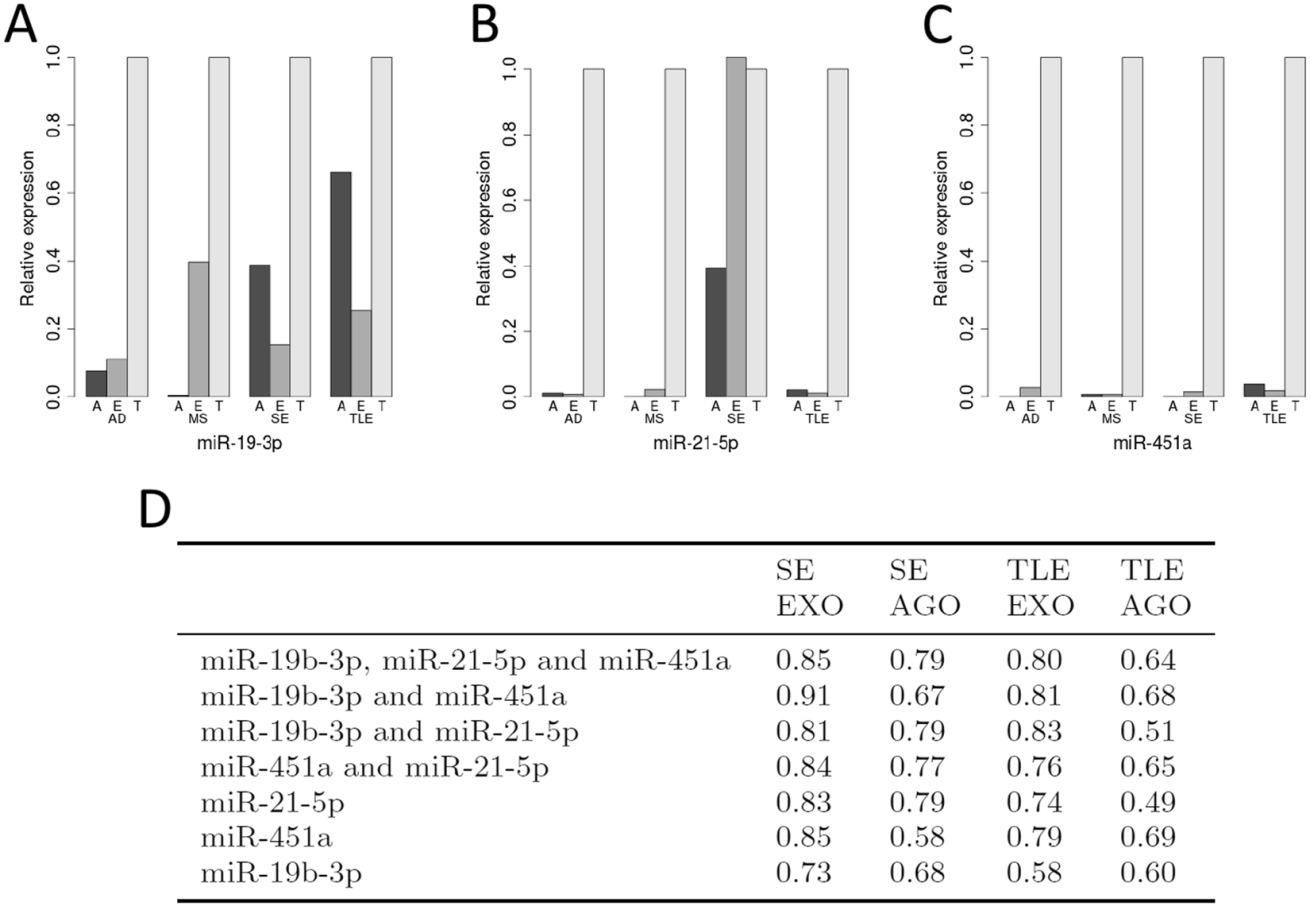
Relative expression of Argonaute2 (**A**), exosome (E) and total (T) microRNA in Alzheimer’s disease (AD), multiple sclerosis (MS), SE and TLE samples. (**D**) AUC following ROC analysis using binomial logistic regression to combine microRNAs for SE samples versus CH, CND and TLE samples, and TLE samples versus CH, CND and SE samples, for Argonaute2 bound microRNA and exosomal microRNA.

The form in which CSF microRNAs are packaged is unknown but could hold additional discriminatory value since unique pathophysiological aspects of neurological disease states such as the activation of astrocytes or microglia relative to neuronal injury and hyperexcitability may influence the release mechanisms and composition of extracellular microRNAs. An assessment of these could provide additional discriminatory value. We therefore separately analysed the levels of miR-19b-3p, miR-21-5p and miR-451a complexed to Argonaute2 or present in exosomes relative to the total microRNA content in CSF (Fig. 5A–C). This analysis revealed a number of important findings: First, microRNAs show a unique pattern of distribution between exosomes and Argonaute2. For miR-19b-3p, the best biomarker for TLE, the majority of microRNA was present in exosomes in Alzheimer’s disease and multiple sclerosis, whereas it tended to be more Argonaute2 bound for TLE and SE. The opposite was found for miR-21-5p, where in SE samples most microRNA was found in exosomes. The abundance of miR-451a was generally low across all TLE samples, with a tendency towards exosomes rather than Argonaute2.

Next, we explored if the form in which the microRNA circulated could provide additional discriminatory value compared to total microRNA. We calculated the AUC following ROC analysis using binomial logistic regression to combine microRNAs for SE samples versus control, other neurological diseases and TLE samples, and TLE samples versus control, other neurological diseases and SE samples, for Argonaute2-bound microRNA and exosomal microRNA. In all cases the AUC was higher when the exosomal microRNA was used versus the Argonaute2 microRNA (Fig. 5D) implying that exosomal microRNA may in some way more accurately reflect the pathophysiology of SE or TLE.

### Correlation of microRNA level in CSF and plasma

We next asked whether levels of the same set of microRNAs in other biofluids would be reflective of CSF microRNA profiles. This is important because there would only be value in measuring CSF microRNAs if they provided diagnostic value above that which could be achieved simply by measuring in blood. To test this idea, we checked for a correlation between the CSF levels of miR-19b-3p, miR-21-5p and miR-451a and levels in plasma collected on the same day from a set of TLE patients.

There was no correlation observed for miR-19b-3p or miR-21-5p between CSF and plasma levels (Supplementary Fig. S6). The levels of miR-451a obtained from plasma showed a trend toward negative correlation with the levels in the same individual in CSF, although this did not reach statistical significance (ρ = −0.75, p = 0.066) (Fig. S6). These findings demonstrate that microRNAs in CSF provide distinct information not available from analysis of plasma.

**Figure 6:**
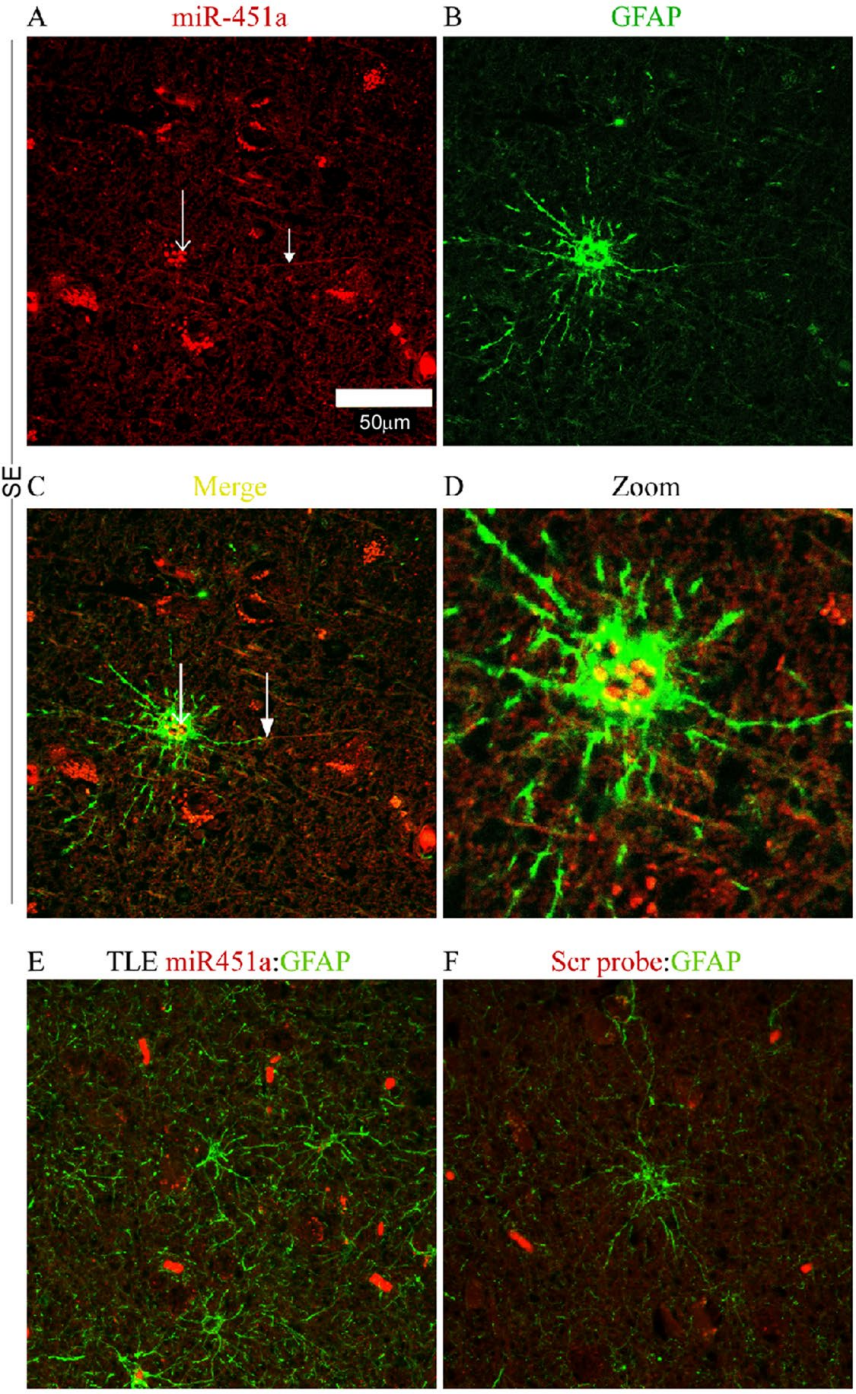
Cellular localization of a microRNA biomarker of SE. Panels show representative *in situ* hybridization for miR-451a in human hippocampal tissue sections from SE and TLE patients. Note astrocyte location of miR-451a in SE tissue whereas expression is low-to-undetectable in TLE samples and in the negative control (microRNA probe omitted).

### Cellular localization of CSF microRNA biomarkers

The cellular distribution of the three microRNAs was investigated using *in situ* hybridization in order to establish whether the microRNA signal was from one or more brain cell type or another source (e.g. blood cell contamination). We detected miR-21-5p and miR-451a in tissue sections from the hippocampus of SE patients (Fig. 6 and data not shown). Staining of both miR-21-5p and miR-451a was observed in astrocyte cell bodies as well as astrocytic processes (for miR-451a) (Fig. 6). In contrast, we were unable to detect miR-451a and miR-21-5p expression in tissue sections obtained from TLE patients (Fig. 6 and data not shown). Efforts to visualize miR-19b in all section types were unsuccessful.

### Pathway links between CSF microRNAs and patho-mechanisms of TLE and SE

**Figure 7:**
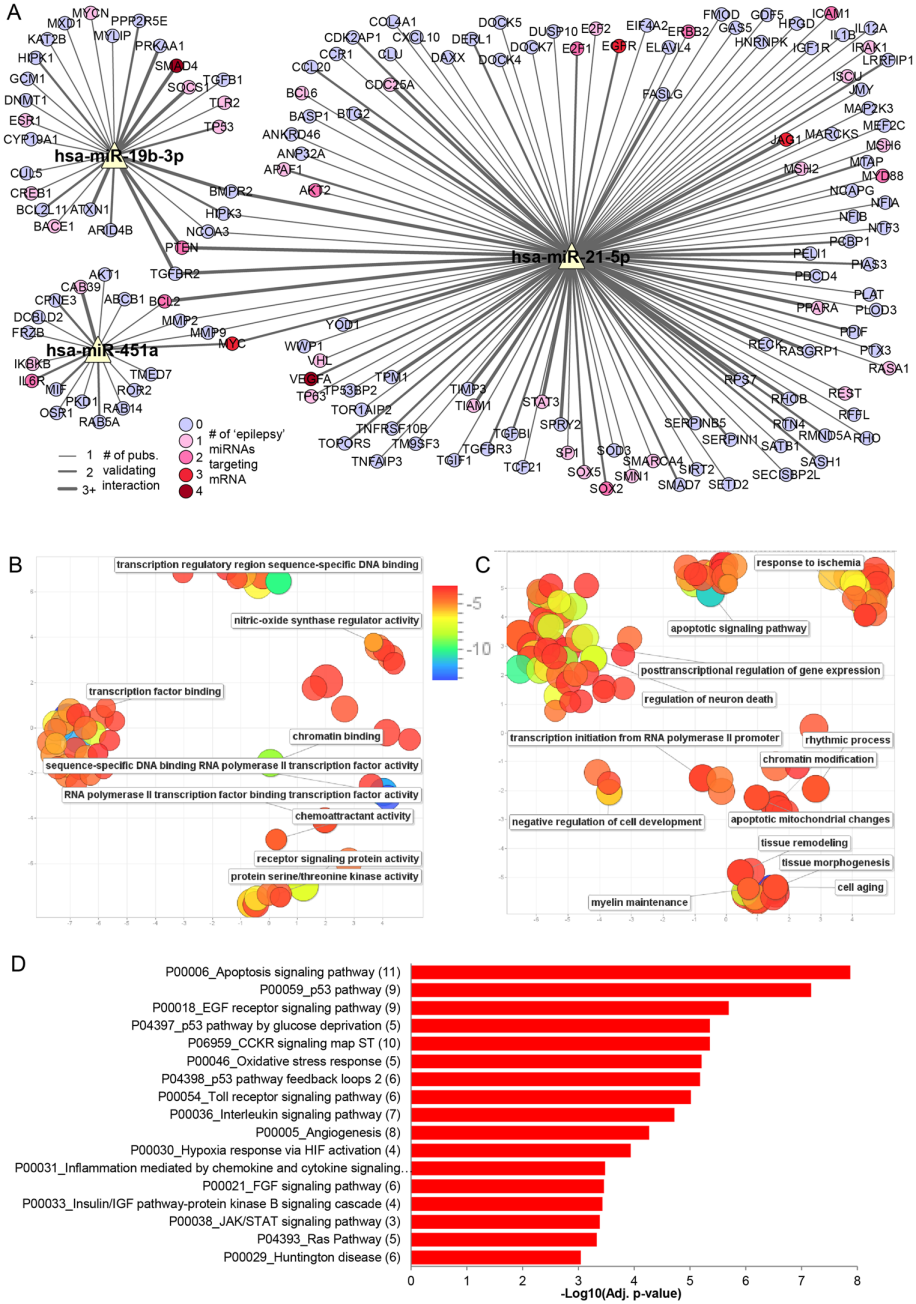
MicroRNA target prediction and pathway enrichment analysis. (**A**) MicroRNA-Target Interaction Network showing targets with strong experimental evidence from miRTarBase for miR-19b-3p, miR-21-5p and miR-451a. Edge thickness corresponds to the number of publications that validated the miRNA-mRNA interaction. Pink/red coloured targets are also targets of one or more of the functionally-validated epilepsy-associated microRNAs (see Materials & Methods). The network was generated using Cytoscape software. (**B**,**C**) Removal of GO term redundancy and semantic visualisation of the significantly associated GO molecular function (**B**) and biological processes (**C**) (adjusted *p*-value < 0.05) using REVIGO. The scatterplot shows the cluster representatives (i.e. terms remaining after the redundancy reduction) in a two dimensional space derived by applying multidimensional scaling to a matrix of the GO terms’ semantic similarities (the axes have no intrinsic meaning). Bubble colour indicates the log_10_(adjusted *p*-value) (legend in upper right-hand corner of **B**); size indicates the frequency of the GO term in the underlying *Homo sapiens* database (bubbles of more general terms are larger). (**D**) Bar plot showing the −log_10_(adjusted *p*-value) of significantly enriched (adjusted *p*-value < 0.001) PANTHER pathways for validated targets of miR-19b-3p, miR-21-5p and miR-451a. The number of targets in a given pathway is shown in parentheses.

Finally, we sought insight into the targets of the microRNAs since this provides additional mechanistic evidence of biomarker potential. We hypothesized that the microRNAs may regulate pathways with known involvement in the pathophysiology of TLE or SE such as relating to neuronal injury or neuroinflammatory or remodelling processes which are strongly activated in models and human studies^24, 55^. Experimentally validated microRNA targets for miR-19b-3p, miR-21-5p and miR-451a were retrieved from miRTarBase^39^. Only human targets with strong evidence were retained (Table S2 and Fig. 7A).

Many of the mRNA targets were found to encode proteins with known or implied roles in TLE or regulated in models of SE. For example, levels of two of the targets of miR-19b-3p, *BCL2L11/BIM* (Bcl-2-like protein 11) and *TP53* (Cellular tumour antigen p53) are altered in experimental and human TLE^56, 57^. Another target, *DNMT1* (DNA (cytosine-5)-methyltransferase 1) regulates DNA methylation, an epigenetic mechanism of gene expression control in human epilepsy^58^.

Targets of miR-21-5p that have been implicated in epilepsy include RE1-silencing transcription factor (REST), which regulates gene expression after status epilepticus^59^, apoptotic protease-activating factor 1 (APAF-1) that contributes to caspase activation during SE^60^, and interleukin-1β and myeloid differentiation primary response protein 88 (MYD88), both involved in inflammatory signalling in epilepsy^61^. Also interesting, the *ABCB1/MDR1* (Multidrug resistance protein 1) is targeted by miR-451a and is associated with pharmacoresistance to antiepileptic drugs^62^. Other targets of miR-451a include anti-apoptotic *AKT1* ^63^ and *BCL-2* ^64^.

We also explored cross-over between the targets of the three CSF microRNAs and a set of microRNAs for which there is functional evidence (*in vivo* modulation) for effects on seizures and/or epilepsy-related pathology (Fig. 7A). This revealed that 42 of the targets of the three CSF microRNAs were also targets of microRNAs functionally validated in epilepsy models.

We uploaded the genes identified as validated targets to Enrichr (gene set enrichment analysis web server)^41^ and explored the gene ontology biological processes, molecular function and pathway involvement of the targets. The significantly associated gene ontology terms (adjusted *p*-value < 0.05) were clustered based on their relatedness and any redundancy was removed^42^. Visualization of these results showed that the largest clusters were formed from the gene ontology biological processes involving posttranscriptional regulation of gene expression (including regulation of neuron death), apoptotic signaling pathways, and tissue morphogenesis and remodeling, and the molecular functions involving transcription factor binding (Fig. 7B,C). Significantly enriched (adjusted *p*-value < 0.001) PANTHER pathways for targets of miR-19b-3p, miR-21-5p and miR-451a are shown in Fig. 7D. A number of potential epilepsy-related pathways were identified including apoptosis signaling pathways, oxidative stress and toll receptor signalling.

Finally, we cross-checked the validated targets against two databases of epilepsy gene mutations: CARPEDB (http://carpedb.ua.edu) and epiGAD^45^. This showed that targets of these microRNAs have been identified with mutations that are known to be associated with different types of epilepsy (Table S3).

## Discussion

The present study found human CSF contains microRNA biomarkers that can support a diagnosis of both TLE and SE. The levels of miR-19b-3p, miR-21-5p and miR-451a were differentially expressed in CSF samples from TLE and/or SE patients compared with control subjects and other neurological diseases. We successfully combined these microRNAs using a logistic regression model which allowed for more specific and sensitive discrimination. Furthermore, models based on microRNA extracted from exosomes were better able to discriminate between samples compared to models based on microRNA bound to Argonaute2. Together, these findings support the potential of microRNAs as diagnostic biomarkers which could provide assistance for differential diagnosis of epilepsy or SE from other neurological diseases.

There is an urgent and unmet need for diagnostic and prognostic biomarkers of epilepsy and SE since clinical examination, electroencephalogram (EEG) and neuroimaging alone is still associated with high rates of misdiagnosis^4, 5^. Much effort to date has been directed towards identifying serum-based protein biomarkers^11, 65^ and more recently serum-based microRNAs^28, 29^, none of which have led to clinical use to date. MicroRNA profiles within circulating blood cells such as leukocytes may also be suitable^66^ although this source of biomarkers has not been explored in either epilepsy or SE. CSF is a potentially more informative source of microRNA than serum or plasma as there is closer contact between brain tissue and fluid and a smaller volume of biofluid into which any microRNAs disperses relative to blood. However, this is balanced against the more invasive procedure needed to obtain the biofluid and CSF is not routinely obtained from epilepsy patients. To our knowledge, the present study is the first report on CSF microRNAs as potential biomarkers of TLE and SE. A number of platform technologies are available for microRNA profiling, each with advantages and disadvantages^67^. Here we used a qPCR-based platform well-suited to profiling low-abundance microRNAs, during the discovery phase to screen microRNAs in individual samples^35^. Although this platform captures a pre-selected number of microRNAs, the coverage is extensive and includes all bona fide human microRNAs^68^. This comes at the expense of sequence specificity^69^ and the ability to identify novel microRNAs such as using RNA sequencing^28, 29^. This necessitates rigorous post-hoc validation of any differentially expressed microRNA. The presence and abundance of microRNA in CSF is still relatively poorly understood compared to plasma or serum and there is not yet consensus as to the number of microRNA that can be expected to be found under normal conditions. The number of microRNA detected is also likely to be influenced by the clinical procedures, profiling methods and the analysis techniques used (i.e. the criteria used to declare microRNAs present or absent, filtering, etc). Recent work suggests numbers of CSF microRNAs range from 50 or less in some studies^32^ to over 400^70^. Other studies have reported detection in the range of 50 to 100 microRNAs on average per sample^71, 72^ suggesting the number of microRNA identified in common-to-all CSF samples in a particular study is often much lower. Our findings, with over 40 microRNAs called present in at least half the samples therefore fall within expected range. Moreover, many of the same microRNA were detected as most abundant have been reported in CSF using other detection platforms^70^. None of the CSF microRNAs were unique compared to our previous findings in human plasma^35^, however the poor correlation between CSF and plasma or serum microRNA levels^70, 73, 74^ suggests that there is potentially an independent origin of the microRNA in CSF compared to plasma/serum.

Despite the relatively low numbers of microRNAs in CSF those that were differentially expressed in TLE or SE was proportionally high. This supports CSF as a good source of biomarkers relative to blood in which the fraction of differentially expressed microRNAs was much smaller relative to those detected^28, 29^. Another advantage of our study was to analyse individual samples during profiling rather than pooling patient samples which may mask important differences^29^. An obvious reason for the higher relative detection of differentially-expressed microRNAs in CSF is proximity to the area of principle pathology. Differences in microRNA levels between neurological diseases may be diluted within the systemic circulation whereas the volume and circulation of CSF may favour biomarker detection.

Clinically, the most interesting comparisons are between TLE or SE and the controls and other neurological diseases. Subtypes of each condition should ideally be possible to differentiate using a biomarker, with the diagnosis of a nonconvulsive SE the most difficult in clinical practice. Here we identified a set of three microRNAs, miR-19b-3p, miR-21-5p and miR-451a, which were differentially expressed in TLE and/or SE compared to controls and other neurological diseases. ROC analysis supported good diagnostic accuracy of these microRNAs, and slightly better than plasma-based microRNAs for discriminating between patients with epilepsy and controls^29^. A unique strength of the present study was the inclusion of CSF from patients with other neurological diseases and we found microRNAs that were different in TLE compared to controls and other neurological diseases (miR-19b-3p) and SE and these groups (miR-451a). Using logistic regression models to combine all three microRNAs we were able to further enhance the discriminatory value of the microRNAs for TLE samples compared to controls, other neurological diseases and SE samples. We also found a combination of miR-21-5p and miR-451a can discriminate SE from TLE, controls and other neurological diseases. Thus, CSF microRNAs, individually or in combination offer potential biomarkers of TLE and SE and discriminate from other neurological diseases. It will be important in future studies to include a larger collection, in particular of nonconvulsive SE samples, and determine whether the same or different microRNAs can identify patients that pose diagnostic challenges. For example, patients with psychogenic non-epileptic attack and other causes of loss of consciousness such as syncope. Samples from such patients were not available for the present study. We also recognize the caveat of using CSF from patients with headache as our controls, the underlying causes of which may also affect results. Samples from additional ethnic groups should also be explored. Anti-seizure drugs and other medications are also potential confounding factors that may influence CSF levels of microRNAs and these were not controlled for in the present study.

The mechanism and cellular origin of the released microRNA in CSF is likely to differ between neurological diseases. In SE it may be due mainly to acute neuronal injury (i.e. lytic release) or reactive gliosis. In TLE where the impact of seizures is less harmful there may be a more controlled release from a mixed population of neurons and reactive glia. MicroRNAs may be intrathecal in origin^75^, or they may originate from brain cells, surrounding brain tissues, or extracranial tissues due to blood- or cerebrospinal-brain barrier disruption associated with seizures^73, 76^. While miR-451a has been reported to be upregulated in brain tissue after seizures and trauma^73, 77, 78^, it is known to be particularly abundant in blood cells^79^. It was important, therefore, to explore the cell types expressing this microRNA. *In situ* hybridization identified miR-451a, as well as miR-21-5p, within astrocytes in SE patients. Given we did not find higher levels of other blood cell-abundant microRNAs in SE samples the increase may reflect upregulation of miR-451a within this cell type or result from uptake from the local environment. Additional experiments will be needed to resolve these possibilities and any pathophysiological significance for astrocyte function. Notably, serum albumin is taken up by astrocytes after SE and this contributes to promoting a hyperexcitable state^80^.

A novel approach in the present study was to investigate the physical form in which microRNAs are present in the CSF. MicroRNAs can be released via passive or active mechanisms and assessing the relative amounts of microRNAs between these systems offers additional diagnostic sensitivity since it is likely the relative glial-neuron-injury contributions differ between neurological diseases due to ischemic components versus degenerative versus inflammatory^81, 82^. Neurons and glia may differ in their capacity to produce exosomes or release Argonaute2-bound microRNA and this may vary across diseases. Here we found high amounts of exosomal miR-19b-3p in CSF from patients with multiple sclerosis, which is characterised by demyelination and glia-mediated neuroinflammatory mechanisms^83^. Microglia are also increasingly recognized for contributions to the pathogenesis of Alzheimer’s disease^84^. In contrast, miR-19b-3p was found to be mostly argonaute-bound in SE and TLE samples. Thus, the underlying pathophysiology may influence the physical form in which extracellular microRNAs circulate and this can be captured to enhance diagnostic insights. The molecular mechanisms that underlie microRNA sorting into microvessicles such as exosomes and their trafficking and receipt by target cells are increasingly understood and thought to represent forms of paracrine signalling. Indeed, the microRNA cargo within these vesicles has been demonstrated to influence gene expression in recipient cells^16^. Whether exosomal microRNAs in CSF have signalling roles or rather reflect the distribution of material from brain tissue in a more indirect way requires further investigation.

Other findings here support the CSF-detected microRNAs as having relevance to the underlying pathophysiology. First, over half of the microRNA have been detected in at least one neural cell type, or brain region, in studies of rodent brains^22, 85, 86^, and a quarter of them are differentially expressed in profiling studies of hippocampal tissue resected from TLE patients^58, 87, 88^. Over 80% of them can be found in EpimiRBase^23^ implying that they have been identified as regulated in at least one epilepsy study in human or rodent. The microRNAs that we have identified here as potential CSF biomarkers, miR-19b-3p, miR-21-5p and miR-451a, have all been previously shown to be up regulated after SE in brain profiling studies of both rat^26, 27, 89–91^ and mouse^92, 93^ and significant up regulation of miR-21-5p has also been shown in the hippocampal tissues of children with TLE^90^ and epileptic adults^27^ compared to controls. Altered expression of miR-21-5p is thought to oppose oedema and mediate anti-apoptotic effects^94^. Furthermore, the significantly associated biological process and pathways of the validated targets of the microRNA were reflective of known processes and pathways in TLE and SE such as tissue morphogenesis, remodelling and cell differentiation, p53 and apoptosis signalling and Toll-like receptor pathways. There was also overlap between targets of the CSF microRNAs and targets of microRNAs which have been functionally validated as affecting brain excitability and seizures. This provides encouraging mechanistic support into their biomarker potential although microRNA-target predictions are heavily biased by the bulk of the validation work being performed in cancer and cell biology fields^95^ and there may be other sources of bias^96^. In contrast, there was little overlap with microRNAs identified as differentially expressed in experimental models of SE or in serum from epilepsy patients with the exception of miR-21-5p which was upregulated in the plasma of rats one week after experimental status epilepticus^25–29^. One of the most frequently identified microRNAs in biofluids in epilepsy studies, miR-146a, was not differentially expressed in the CSF of TLE or SE patients. These findings fit with our observation that plasma levels of the three microRNAs showed no correlation with CSF levels and emphasise that microRNA biomarkers are specific to the analysed biofluid.

In conclusion, the present study identifies CSF microRNAs as potential biomarkers of TLE and SE. Analyses of the physical form in which the microRNAs circulate provide further diagnostic insight and the targets of the microRNAs and the pathways in which they operate suggest plausible mechanistic links to known pathomechanisms. These findings should be confirmed in other patient cohorts. Future studies could investigate whether the combination of regulated microRNA from CSF and blood further increases specificity as biomarkers and whether microRNA levels in focal epilepsies differ from genetic or generalized epilepsies. The findings should provide opportunities to replicate and extend the findings and could lead toward the development of a molecular biomarker for the diagnosis of epilepsy, SE or other neurological disorders. Finally, microRNAs represent only one among many different species of non-coding RNA with brain functions^97^. Other short as well as long noncoding RNA species, if detectable in CSF, may also serve as suitable molecular biomarkers of TLE or SE.

### Ethics approval and consent to participate

Clinical samples were obtained in accordance with the Declaration of Helsinki and ethical approval was obtained from the local medical ethics committees at the University of Magdeburg, The Philipps University of Marburg and The Friedrich-Alexander-University Erlangen-Nurnberg. This included informed written consent given by patients or their legal representatives if the patients were obtunded.

## Electronic supplementary material

Supplementary Information file
