# Supplementary material for: Cerebrospinal fluid microRNAs are potential biomarkers of temporal lobe epilepsy and status epilepticus
Source: Sci Rep. 2017 Jun 12;7:3328. doi: 10.1038/s41598-017-02969-6 (PMC5468228; doi:10.1038/s41598-017-02969-6)
Supplement: Supplementary file 1 — Supplementary Information file [file 41598_2017_2969_MOESM1_ESM.pdf]

Supplementary Information

Cerebrospinal fluid microRNAs are potential biomarkers of temporal lobe epilepsy and status epilepticus

Rana Raoof, Eva M. Jimenez-Mateos, Sebastian Bauer, Björn Tackenberg, Felix Rosenow, Johannes Lang, Müjgan Dogan Onugoren, Hajo Hamer, Tessa Huchtemann, Peter Körtvélyessy, Niamh M. C. Connolly, Shona Pfeiffer, Jochen H.M. Prehn, Michael A. Farrell, Donncha F. O'Brien, David C. Henshall and Catherine Mooney

Supplementary Materials and Methods

RNA extraction

Total RNA was extracted from 200 μl of CSF or plasma using miRCURY^{TM} RNA Isolation Kit (Exiqon) according to the manufacturer protocol. Lysis of membranized particles within the sample were performed using lysis solution. After a protein precipitation step the resultant supernatant was collected and mixed with isopropanol then loaded into a spin-column which binds only the RNA. This was followed by two respective wash steps followed by a centrifugation for 2 minutes at 11,000 x g to dry the column membrane completely. The final purified RNA was eluted in 25 μl RNAase free water. Small RNA and microRNA concentration and microRNA % in each sample was assessed using a fragment analyzer (Advanced Analytical).

MicroRNA expression profiling

MicroRNA profiling was performed using the OpenArray platform from Applied Biosystem, as described (Mooney *et al.*, 2015). The OpenArray reverse transcription reaction was performed according to the manufacturer's protocol using 3 μl of total RNA in a mix of 0.75 μl Megaplex RT primer pools (human Pools A or B Cat No. 4444750) from Applied Biosystem, 1.5 μM dNTPs with dTTPs, 75U Multiscribe Reverse Transcriptase, 1X RT Buffer, 1.5 μM MgCl2, 1.8U RNAase inhibitor (RT kit Cat No 4366596, AB). Reverse transcription reaction was performed in Applied Biosystem thermal cycler.

To increase the quantity of desired cDNA before performing PCR and to significantly increase the ability to detect low abundance transcripts, a pre-amplification step was performed according to the manufacturer's recommendation. 2.5 μl RT product was mixed with 1X Megaplex PreAmp primers (10X Human Pool A and B Cat. No. 4444748, AB), 1X TaqMan PreAmp master mix (2X, Cat No. 4391128, AB). Pre-amplification reaction was performed in an Applied Biosystem thermal cycler.

PreAmp product was first diluted with 0.1X TE to a ratio of 1:40, 22.5 μl of diluted PreAmp product was then added to same volume of 2X TaqMan OpenArray Real time PCR Master Mix (Cat No. 4462164, AB) in the 384-well OpenArray sample loading plate. The manufacturers protocol was followed and the OpenArray panels were automatically loaded by the OpenArray AccuFill System. Each panel enables the quantification of microRNA expression in three samples and up to four panels can be cycled simultaneously, allowing for the analysis of 12 samples on a QuantStudio 12K Flex Real-Time PCR system. 754 human microRNAs were amplified in each sample together with 16 replicates each of four internal controls (ath-miR159a, RNU48, RNU44 and U6 rRNA).

Reverse Transcription and Real-Time PCR validation

Validation of OpenArray findings was performed using the small-scale RT-qPCR protocol adapted from (Mitchell *et al.*, 2008). RNA was extracted from CSF samples using miRCURY RNA isolation kit-biofluid. For the Reverse Transcription step, a master mix containing, 1x RT buffer (AB), 1.26 units RNAase inhibitor (AB), 16.5 units Multiscribe reverse transcriptase enzyme (AB), 0.025 mM dNTPs (AB), 0.6 μl of the specific stem loop RT primer for each microRNA was prepared and added to 1.7 μl of total RNA (similar to Mitchell *et al.* (2008)). For real-time PCR amplification, cDNA was diluted in a ratio of 1:6.5 and assayed using 1X TaqMan Fast Universal PCR Master Mix (AB) and microRNA-specific PCR primers. The amplification was done in triplicate and a negative control was included for each primer. The same protocol was followed to quantify the microRNA levels of miR-19b-3p, miR-21-5p and miR-451a in plasma samples from TLE patients.

Analysis of microRNA content within exosomes and complexed to Argonaute2

To indicate whether significantly differentially expressed microRNAs are protein bound (Argonaute2) or enclosed within exosomes, we pooled the CSF samples into five TLE samples, five SE samples, three multiple sclerosis samples and three Alzheimer's disease samples. Each pool was then divided into three parts: 300 μl was allocated for exosome precipitation; 300μl for Argonaute2 immunoprecipitation; and 200 μl for total RNA extraction.

Exosome precipitation was performed using the ExoQuickTM Exosome Precipitation Solution (System Biosciences-SBI) (Lobb *et al.*, 2015). Briefly, 75.6 μl of precipitation solution was added to 300 μl of CSF. The mix was incubated for 24 hours at $4°C$, followed by centrifugation at 1500 x g for 30 minutes. After centrifugation the exosomes appear as a white pellet at the bottom of the tube. To remove any excess precipitation solution a second quick centrifugation step at 1500 x g for 5 minutes was performed. Total RNA was then extracted from exosomes using miRCURY RNA isolation kit-biofluid according to manufacturer protocol.

Argonaute2 pull-down from CSF was adapted from (Jimenez-Mateos *et al.*, 2012) as follows. 300 μl of pooled CSF was incubated overnight with 10 μg of antibodies against Argonaute-2 (C34C6, Cell Signaling Technology) at $4°C$. Protein A-agarose beads (Santa Cruz Biotechnology) were added, mixed and incubated for 4 hours at $4°C$, then centrifuged and the supernatant removed. The pellet was washed three times with immunoprecipitation buffer containing 300 mM NaCL, 5 mM MgCl2, 0.1% NP-40, 50 mM Tris HCl. Total RNA was extracted from Argonaute2 IP pellet using 200 μl of Trizol reagent (Invitrogen). Phase separation was performed using 50 μl of chloroform, the upper aqueous phase was collected and transformed into a new tube.

Total RNA in the sample was precipitated and washed using Isopropanol and 75% ethanol respectively. The final RNA pellet was dissolved in 12 μl RNAase free water.

MicroRNA expression after exosome precipitation and Argonaute2 IP

For the reverse transcription reaction, RT primers for miR-19b-3p, miR-21-5p and miR-451a were pooled and diluted with 1X TE buffer to a final concentration of 0.05X. An RT mix was prepared containing 3 μl Multiscribe reverse transcriptase enzyme, 1.5 μl 10X RT buffer, 0.19 μl RNAase inhibitor, 0.3 μl dNTPs, 6 μl RT primer pool and 1.01 μl dH2O. For a 15 μl RT reaction, 3 μl RNA was mixed with 12 μl RT mix.

To increase the quantity of desired cDNA before performing PCR, a pre-amplification step was performed for each sample. Pre-amplification reaction mix was prepared by pooling the TaqMan microRNA assays of the above microRNAs and diluting it with 1X TE buffer to a final concentration of 0.2X. 3.75 μl of preamp primer pool was added to 2.5 μl RT product and 12.5 μl of 2X TaqMan preamp master mix, dH_2O was added so the final volume of the reaction is 25 μl. RT-qPCR was then performed in 96 well plate following the same protocol described earlier.

Supplementary Figures S1 - 6

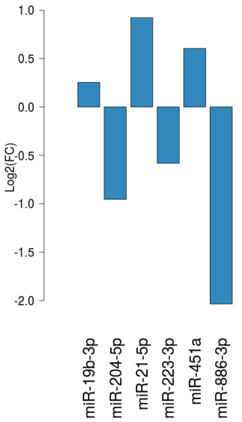

(a) CND-CH

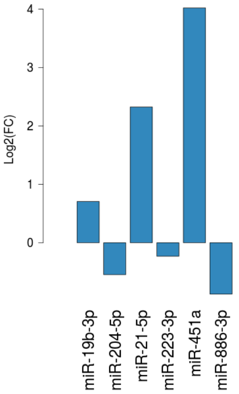

(b) SE-CH

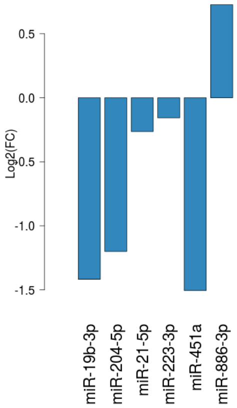

(c) TLE-CH

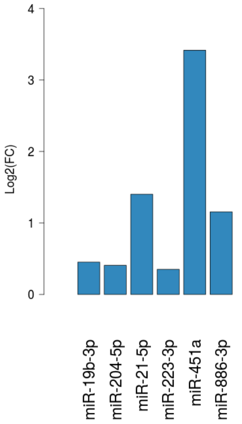

(d) SE-CND

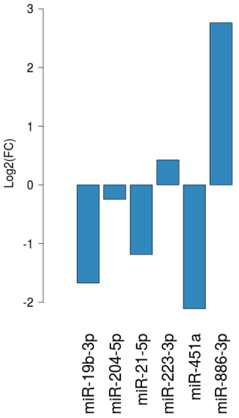

(e) TLE-CND

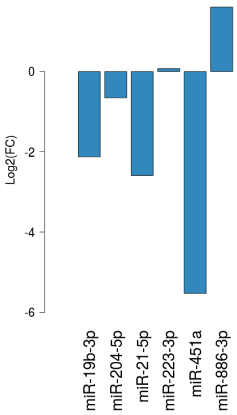

(f) TLE-SE

Figure S1: **Validation of microRNA.** Barplots showing the fold change (FC) in the first indicated group when compared to the second (i.e. TLE-CH is the FC in TLE compared to CH) following Taqman individual microRNA assays of miR-19b-3p, miR-21-5p, miR-204-5p, miR-223-3p, miR-451a and miR-886-3p for each of the four groups: CH (N = 25), CND (N = 25), SE (N = 16) and TLE (N = 14). The fold change is calculated as $FC = 2^{-\Delta\Delta Ct}$, where $\Delta\Delta Ct = Ct_{miRNA} - Ct_{miR-24}$.

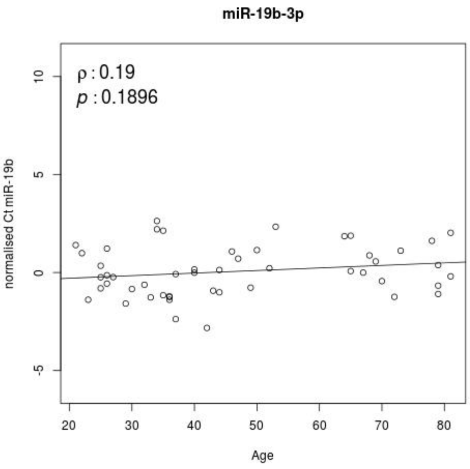

(a) miR-19b-3p

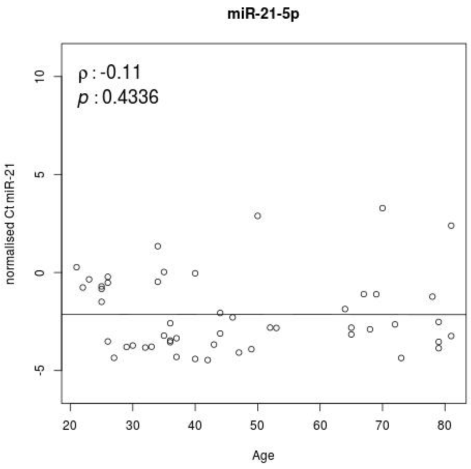

(b) miR-21-5p

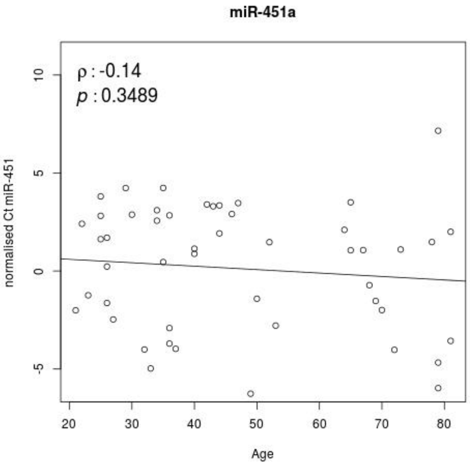

(c) miR-451a

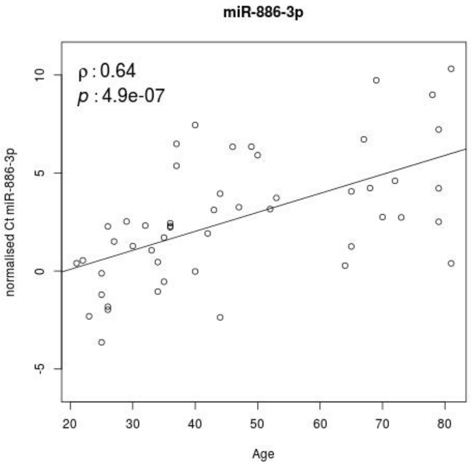

(d) miR-886-3p

Figure S2: **Correlation between normalised Ct of microRNA in CSF and patient age in controls.**

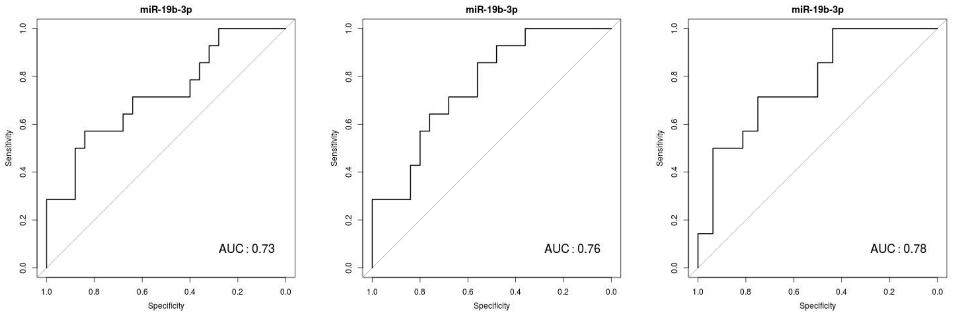

(a) TLE-CH (b) TLE-CND (c) TLE-SE

Figure S3: **ROC analysis for miR-19b-3p in (a) TLE versus CH (b) TLE versus CND (c) TLE versus SE.**

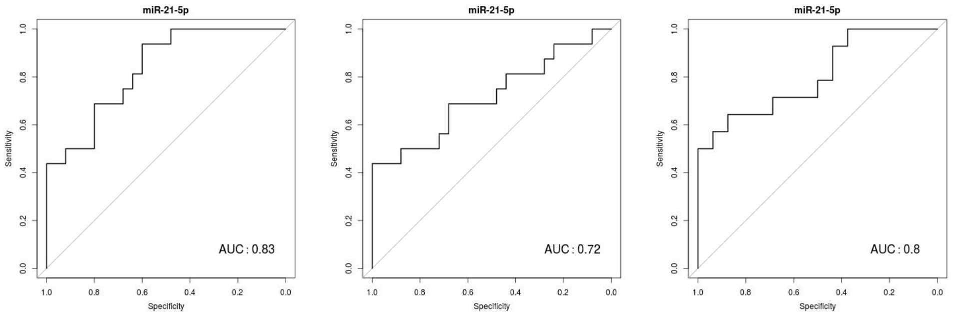

(a) TLE-CH (b) TLE-CND (c) SE-TLE

Figure S4: **ROC analysis for miR-21-5p in (a) SE versus CH (b) SE versus CND (c) SE versus TLE.**

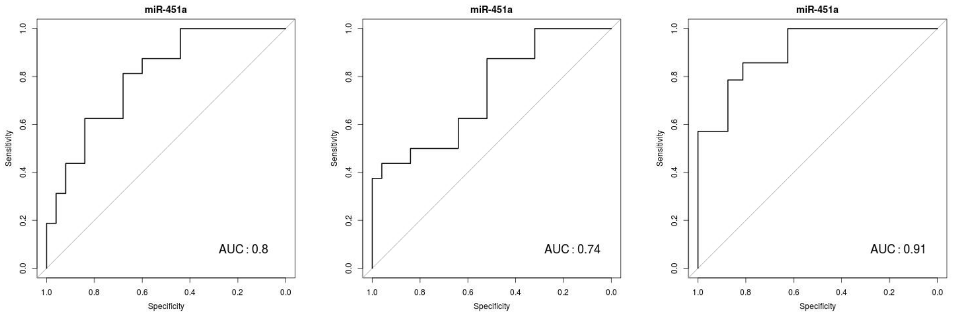

(a) TLE-CH (b) TLE-CND (c) SE-TLE

Figure S5: **ROC analysis for miR-451a in (a) SE versus CH (b) SE versus CND (c) SE versus TLE.**

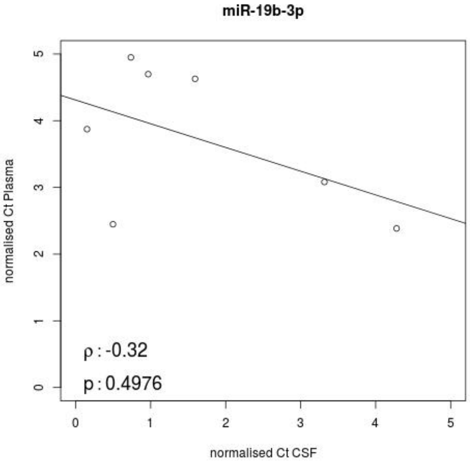

(a) miR-19b-3p

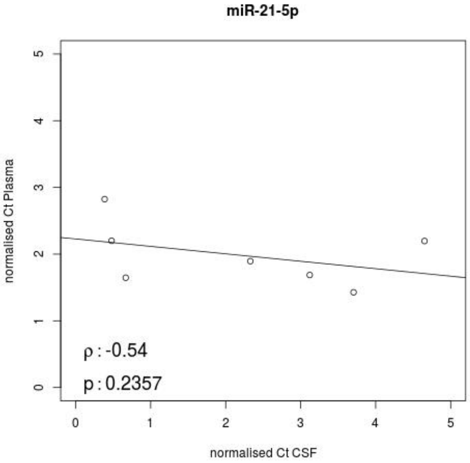

(b) miR-21-5p

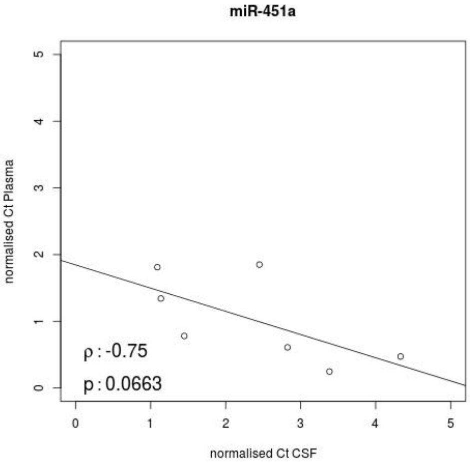

(c) miR-451a

Figure S6: **Correlation of normalised Ct between CSF and plasma for (a) miR-19b-3p, (b) miR-21-5p and (c) miR-451a.**

Supplementary Tables S1-3

Table S1: **Demographics and clinical data for TLE, SE, CND and CH samples.**

Control-Chronic Headache			
Sex	Age	Diagnosis	Experiment
F	25	chronic headache	OA, P, A/E
F	25	chronic headache	OA, P, A/E
F	26	chronic headache	OA, P, A/E
F	32	chronic headache	P
F	35	chronic headache	P
F	36	chronic headache	P
F	37	chronic headache	P
F	40	chronic headache	OA, P
F	40	chronic headache	P, A/E
F	43	chronic headache	OA, A/E
F	44	chronic headache	OA, P
F	44	chronic headache	P, A/E
F	46	chronic headache	P
F	47	chronic headache	P, A/E
F	48	chronic headache	OA
F	49	chronic headache	P
F	50	chronic headache	P
F	52	chronic headache	P
M	21	chronic headache	OA,P, A/E
M	22	chronic headache	P
M	23	chronic headache	OA, P, A/E
M	25	chronic headache	OA, P, A/E
M	26	chronic headache	OA, P, A/E
M	34	chronic headache	OA, P, A/E
M	34	chronic headache	OA, P, A/E
M	35	chronic headache	OA, P, A/E
M	37	chronic headache	P
M	41	chronic headache	OA, A/E
Control-Neurological Diseases			
Sex	Age	Diagnosis	Experiment
F	67	Alzheimer's disease	P, A/E
F	70	Alzheimer's disease	P, A/E
F	79	Alzheimer's disease	P, A/E
M	65	Alzheimer's disease	P, A/E
M	68	Alzheimer's disease	P, A/E
M	69	Alzheimer's disease	P, A/E
M	78	Alzheimer's disease	P, A/E
M	79	Alzheimer's disease	P, A/E
M	81	Alzheimer's disease	P, A/E
F	26	multiple sclerosis	P
F	27	multiple sclerosis	P, A/E
F	29	multiple sclerosis	P, A/E
F	30	multiple sclerosis	P, A/E
F	33	multiple sclerosis	P, A/E
F	36	multiple sclerosis	P, A/E
F	36	multiple sclerosis	P, A/E
F	42	multiple sclerosis	P, A/E
M	43	multiple sclerosis	P, A/E
M	81	multiple sclerosis	P, A/E

F	53	metastatic brain tumour (breast cancer)	P
F	64	undefined gait disorder	P
F	65	sinus vein thrombosis	P
M	72	choroidal melanoma (with possible brain metastasis)	P
M	73	motor neuron disease	P
M	79	right hypoglossal paresis	P

TLE

Sex	Age	Diagnosis	Experiment
F	40	TLE right	OA, P, A/E
F	40	TLE+HS	OA, A/E
F	42	focal	OA, P, A/E
F	45	TLE	OA, P, A/E
F	55	TLE	OA, P, A/E
F	56	multifocal epilepsy	OA, P, A/E
M	18	TLE right	OA, P, A/E
M	20	TLE	OA, P, A/E
M	23	TLE	OA, P, A/E
M	27	TLE	OA, P, A/E
M	34	multifocal epilepsy	OA, P, A/E
M	35	TLE	OA, P, A/E
M	48	TLE	OA, P, A/E
M	57	TLE	OA, P, A/E
M	60	TLE left	OA, P, A/E

SE

Sex	Age	Semiology	Aetiology	Experiment
F	58	FSE	encephalopathy of unknown origin	OA, P, A/E
F	59	FSE	undetermined	OA, P, A/E
F	76	FSE	post-traumatic subarachnoid haemorrhage	OA, P, A/E
F	78	FSE	brain tumour resection in 1990	P
F	81	FSE	undetermined	P
F	85	FSE	left temporal lobe tumour	OA, P, A/E
F	53	NCSE	undetermined	P
F	74	NCSE	undetermined	OA, P, A/E
M	60	FSE	alcohol abuse/hypoglycaemia IDDM	OA, P, A/E
M	61	FSE	aneurysmal bleeding	OA, P, A/E
M	77	FSE	immediate SE after stroke	OA, P, A/E
M	91	FSE	apoplexy	OA, P, A/E
M	67	GTC	HIV-encephalitis/schizophrenia	OA, P, A/E
M	78	GTC	lung tumour with brain metastases	OA, A/E
M	83	GTC	dementia	OA, P, A/E
M	53	NCSE	multiple sclerosis	OA, P, A/E
M	63	NCSE	alcohol withdrawal	OA, P, A/E
M	88	NCSE	post-stroke	OA, P, A/E

Key: M = Male; F = Female; FSE = Focal SE; GTC = generalized tonic clonic; NCSE = non convulsive SE; insulin dependent diabetes mellitus (IDDM); DD= differential diagnosis; OA = samples used for profiling; P = samples used in RT-qPCR validation; A/E = samples used for Ago/Exo analysis.

Table S2: **Validated targets for miR-21-5p, miR-19b-3p and miR-451a from miR-TarBase (Chou *et al.*, 2016).**

miR-21-5p	miR-21-5p	miR-19b-3p	miR-451a
AKT2	NFIB	ARID4B	ABCB1
ANKRD46	NTF3	ATXN1	AKT1
ANP32A	PCBP1	BACE1	BCL2
APAF1	PDCD4	BCL2L11	CAB39
BASP1	PELI1	BMPR2	CPNE3
BCL2	PIAS3	CREB1	DCBLD2
BCL6	PLAT	CUL5	FRZB
BMPR2	PLOD3	CYP19A1	IKBKB
BTG2	PPARA	DNMT1	IL6R
CCL20	PPIF	ESR1	MIF
CCR1	PTEN	GCM1	MMP2
CDC25A	PTX3	HIPK1	MMP9
CDK2AP1	RASA1	HIPK3	MYC
CLU	RASGRP1	KAT2B	OSR1
COL4A1	RECK	MXD1	PKD1
CXCL10	REST	MYCN	RAB14
DAXX	RFFL	MYLIP	RAB5A
DERL1	RHO	NCOA3	ROR2
DOCK4	RHOB	PPP2R5E	TMED7
DOCK5	RMND5A	PRKAA1	
DOCK7	RPS7	PTEN	
DUSP10	RTN4	SMAD4	
E2F1	SASH1	SOCS1	
E2F2	SATB1	TGFB1	
EGFR	SECISBP2L	TGFBR2	
EIF4A2	SERPINB5	TLR2	
ELAVL4	SERPINI1	TP53	
ERBB2	SETD2		
FASLG	SIRT2		
FMOD	SMAD7		
GAS5	SMARCA4		
GDF5	SMN1		
HIPK3	SOD3		
HNRNPK	SOX2		
HPGD	SOX5		
ICAM1	SP1		
IGF1R	SPRY2		
IL12A	STAT3		
IL1B	TCF21		
IRAK1	TGFBI		
ISCU	TGFBR2		
JAG1	TGFBR3		
JMY	TGIF1		
LRRFIP1	TIAM1		
MAP2K3	TIMP3		
MARCKS	TM9SF3		
MEF2C	TNFAIP3		
MMP2	TNFRSF10B		
MMP9	TOPORS		
MSH2	TOR1AIP2		
MSH6	TP53BP2		
MTAP	TP63		
MYC	TPM1		
MYD88	VEGFA		
NCAPG	VHL		
NCOA3	WWP1		
NFIA	YOD1		

Table S3: **Validated targets for miR-19b-3p, miR-21-5p and miR-451a from miR-TarBase with genes in the CARPEDB (http://carpedb.ua.edu/search.cfm) and epiGAD (Tan and Berkovic, 2010) databases.**

Epilesy gene database	Gene
miR-19b-3p	
egad	ARC
egad	MTRR
carpedb	NF1
egad	NR3C1
carpedb	SLC6A8
egad	SLC9A1/NHE1
carpedb	SLC9A6
carpedb	TCF4
miR-21-5p	
carpedb	ADNP
carpedb	EIF2S1
carpedb	EPM2A
carpedb	FMR1
carpedb, egad	IL1B
carpedb	PLAT
carpedb	PLD1
carpedb	ZNF354A
miR-451a	
carpedb, egad	ABCB1

References

Chou CH, Chang NW, Shrestha S, Hsu SD, Lin YL, Lee WH, *et al.* miRTarBase 2016: updates to the experimentally validated miRNA-target interactions database. Nucleic acids research 2016; 44: D239–D247.

Jimenez-Mateos EM, Engel T, Merino-Serrais P, McKiernan RC, Tanaka K, Mouri G, *et al.* Silencing microRNA-134 produces neuroprotective and prolonged seizure-suppressive effects. Nature Medicine 2012; 18: 1087–1094.

Lobb RJ, Becker M, Wen SW, Wong CS, Wiegmans AP, Leimgruber A, *et al.* Optimized exosome isolation protocol for cell culture supernatant and human plasma. Journal of Extracellular Vesicles 2015; 4: 27031–27031.

Mitchell PS, Parkin RK, Kroh EM, Fritz BR, Wyman SK, Pogosova-Agadjanyan EL, *et al.* Circulating microRNAs as stable blood-based markers for cancer detection. Proceedings of the National Academy of Sciences 2008; 105: 10513–10518.

Mooney C, Raoof R, El-Naggar H, Sanz-Rodriguez A, Jimenez-Mateos EM, and Henshall DC. High Throughput qPCR Expression Profiling of Circulating MicroRNAs Reveals Minimal Sex-and Sample Timing-Related Variation in Plasma of Healthy Volunteers. PloS one 2015; 10: e0145316.

Tan NC and Berkovic SF. The Epilepsy Genetic Association Database (epiGAD): analysis of 165 genetic association studies, 1996–2008. Epilepsia 2010; 51: 686–689.
